# Supplementary material for: Complexome profiling of the Chlamydomonas psb28 mutant reveals TEF5 as an early PSII assembly factor
Source: Plant Cell. 2025 Mar 17;37(6):koaf055. doi: 10.1093/plcell/koaf055 (PMC12164590; doi:10.1093/plcell/koaf055)
Supplement: koaf055_Supplementary_Data [file koaf055_supplementary_data.zip › psb28_interactive.zip!/plots/intensity/qualityPlots/peptides/TEF29_Cre01.g007850.t1.2.html]

### Cre01.g007850.t1.2

Trivial name: TEF29  
SeqCoverage [median]: 22.10  
Mass: 17.09 kDa  
  
 

| Sequence | MissedCleavage | Mass [Da] | wtIntensity | muIntensity | Score |
| --- | --- | --- | --- | --- | --- |
| DVLVQR | 0 | 728.42 | 18755.06 | 18623.22 | 113.82 |
| HDFSAPDFK | 0 | 1062.48 | 8004.33 | 6306.37 | 171.73 |
| HDFSAPDFKEVK | 1 | 1418.68 | 1373.33 | 1790.02 | 45.83 |
| HDFSAPDFKEVKDR | 2 | 1689.81 | 1550.45 | 3243.51 | 92.04 |
| MPMVPQQYPAEVR | 0 | 1544.75 | 5549.01 | 4754.70 | 149.96 |
|  |  |  | 35232.2 | 34717.8 |  |
